# Uncommon Association of Two Anatomical Variants of Cerebral Circulation: A Fetal-Type Posterior Cerebral Artery and Inferred Artery of Percheron, Complicated with Paramedian Thalamomesencephalic Stroke—Case Presentation and Literature Review

**DOI:** 10.1155/2018/4567206

**Published:** 2018-09-24

**Authors:** Aurelian Anghelescu

**Affiliations:** ^1^Neurorehabilitation Clinic, Teaching Emergency Hospital “Bagdasar-Arseni”, Romania; ^2^“Carol Davila” University of Medicine and Pharmacy, Bucharest, Romania

## Abstract

**Background:**

The unilateral fetal variant of the posterior cerebral artery (FPCA) is characterized by the congenital absence of the P1 arterial segment. The artery of Percheron (AOP) is an uncommon vascular variant, in which a single dominant thalamoperforating arterial trunk arises from one P1 segment, bifurcates, and provides bilateral supply to the paramedian thalami and rostral midbrain.

**Case Presentation:**

This is a retrospective case study of a 37-year-old man with multiple lifestyle risk factors (chronic marijuana and tobacco abuse), who suffered a thalamomesencephalic stroke, rapidly worsening to comatose state. After restoration of consciousness, he clinically manifested with left paramedian midbrain syndrome. Imaging demonstrated an asymmetric paramedian thalamic infarction with mesencephalon extension, patency of the basilar, vertebral arteries, and left PCA and right-sided FPCA, respectively. Left-sided thalamoperforating arterioles were not differentiated; AOP was inferred. Neither evident clinical source of embolus nor prothrombotic states were found. Mobile cardiac telemetry and transesophageal echocardiography were not available. The diagnosis was established too late for thrombolytic treatment. Anticoagulation was indicated during the acute and subacute stages, followed by low dose of antiplatelet.

**Discussion:**

This uncommon cerebrovascular configuration (FPCA+AOP) might be the fourth case described in the literature. Sustained rehabilitation and abstinence from tobacco and cannabis led to favorable outcomes.

## 1. Background

The vascular anatomy of the posterior circulation is complex and variable. Autopsy studies and structural imaging scans have detected normal anatomical variations in the morphology of the circle of Willis (incomplete circle, asymmetrical with duplication, absence or fusion of components, and fenestrations), are present in 48–58% of the general population [1–4], and arise during fetal development.

The distinctive landmark of the fetal (origin of the) posterior cerebral artery (FPCA) refers to the precommunicating arterial segment (P1), which may be congenitally absent or markedly hypoplastic (uni- or bilaterally) [1–6]. In this fetal-type posterior circle of Willis, PCA originates directly from the ipsilateral internal carotid (ICA), with no connection with the basilar artery. Structural imaging scans demonstrated that complete agenesis of P1 and absence of the posterior communicating artery (PCoA) may be found unilaterally (in 4–26% subjects) or bilaterally (in 2–4% cases) [1, 2, 5, 6].

Studies of human cadaveric brains demonstrated four major thalamic arterial territories (anterior, paramedian, inferolateral, and posterior), with significant variations and overlaps in vascular irrigation. These territories are supplied by the polar (originating from the PCoA), paramedian/thalamoperforating arteries (TPAs), emerging from the P1 segment, thalamogeniculate (emerging from the P2 segment of the PCA), and posterior choroidal arteries [7–17].

Posterior TPAs arise from the superior, posterior, or posterosuperior surfaces of P1. Their morphological diversity and branching patterns, with multiple variations and complex courses, were subject of autopsy studies [7–17] and structural imaging scans [18–37], respectively.

These perforating arteries are of utmost importance and irrigate the posterior part of the thalamus, medial ventral thalami, the walls of the third ventricle, hypothalamus, and subthalamic–mesencephalic junctions (subthalamus, substantia nigra, red nucleus, oculomotor nucleus, trochlear nucleus, reticular formation of the midbrain, pretectum, rhomboid fossa, and the posterior part of the internal capsule) [7–17].

Artery of Percheron (AOP) is a normal, but uncommon vascular variant of the paramedian branches of the PCA in which a single dominant TPA trunk arises from one P1 segment, bifurcates, and provides bilateral supply to the paramedian thalami and the rostral midbrain [7, 8]. Occlusion of this uncommon arteriole results in a characteristic pattern of bilateral thalamic infarcts with or without mesencephalic involvement.

## 2. Case Presentation

This is a retrospective case study of a slim 37-year-old man exhibiting associated lifestyle risk factors (chronic marijuana and tobacco abuse, but neither alcohol excess, nor other illicit drugs) who suffered an acute thalamomesencephalic stroke, rapidly worsening to a comatose state.

Familial and personal medical history was negative for associated cardiocerebrovascular pathology or other specific risk factors.

In the evening that preceded the cerebral infarction, he submitted a large and elaborate tattoo over the left hypochondrium and abdominal (lumbar) flank and smoked a few cigarettes with cannabis.

The following morning, he experienced acute onset of dizziness, visual, speech, and gait disturbances.

He was admitted to the emergency room with walking difficulties, disturbed balance and coordination of movements, slurred speech, diplopia, confusion, and left palpebral ptosis. Neurological examination revealed right-sided severe ataxic hemiparesis, dysarthria, left palpebral ptosis and mydriasis, divergent strabismus, and fluctuating consciousness (Glasgow coma scale, GCS 10/15).

Blood tests (white blood cells count, hemoglobin, electrolytes, liver, and renal function) revealed normal results. Urine toxicology at admission was positive only for tetrahydrocannabinol; no other illicit drugs were present on tox screen. Electrocardiogram (EKG) and chest X-ray findings were normal. Clotting tests were normal [antithrombin III was 108% (>80%), homocysteine was 7.5 *μ*mol (≤ 12), lupus anticoagulant was negative, antinuclear antibodies were 0.3 UM (<0.7), C protein was 117% (70-130)].

Emergent computed tomography (CT) scan on the day of admission showed no gross abnormality and no evidence of cerebral hemorrhage or encephalitis.

In a few hours he become comatose (GCS 7/15) and was transferred to the intensive therapy unit. Intubation and ventilation support were not necessary. EKG monitoring during admission in the intensive care unit did not revealed pathological aspects.

**Figure 1:**
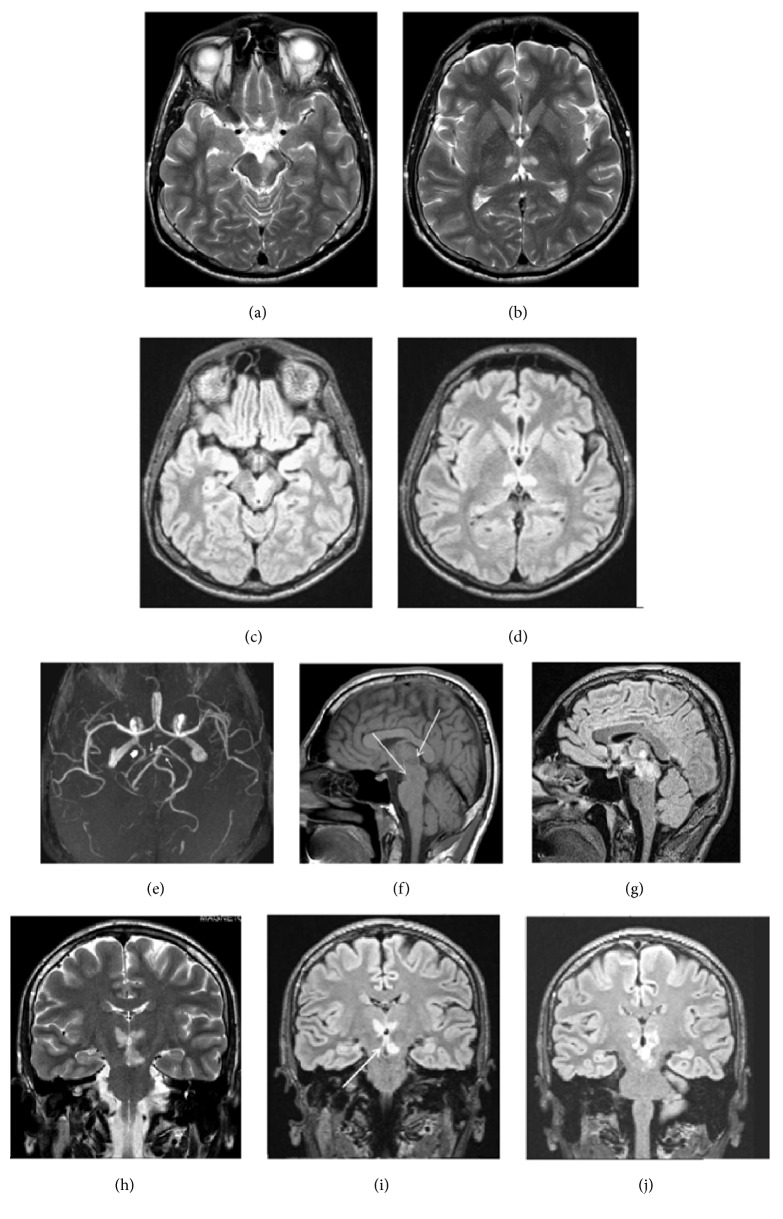
(a) Axial sections of T2-weighted images obtained 20 hours after the onset of symptoms showed areas of increased signal intensity in the left mesencephalon and (b) paramedian thalamic nuclei. (c, d) Axial brain FLAIR images. Hyperintense signals in the rostral midbrain and the paramedian thalamic suggest acute Percheron artery infarction. (c) “V-shaped” hyperintense signal along the pial surface of interpeduncular fossa in midbrain. The mesencephalon lesion extends to the periaqueductal gray matter. (e) MR angiography shows a right-sided FPCA (thick white arrow), arising directly from the ipsilateral internal carotid artery. Patency of the basilar artery and tip, left PCA, and posterior communicating artery; the thin arrows indicate the superior cerebellar arteries, with normal appearance. (f) Sagittal section, T1-weighted: showed ill-defined areas of hypodensity in the thalamopeduncular junction (white arrows). (g) Sagittal section, FLAIR: hyperintense images with the same topography. Coronal sections on T2 (h) and FLAIR images (i, j). Relatively symmetric hyperintense signals in the paramedian inferior thalami, extending (asymmetrically) into the medial and rostral mesencephalon (territory of the artery of Percheron). (i) Coronal section, FLAIR: “lambda-shaped” (Λ) hyperintense signal, adjacent to the pial layer of the interpeduncular fossa, next to the infarction zones in the thalamic–mesencephalon junction, equivalent version of the “V-shape” observed in axial sections (c). (FLAIR, fluid-attenuation inversion recovery images; FPCA, fetal posterior cerebral artery.)

At about 20 hours after the onset of stroke, magnetic resonance imaging (MRI) of the brain and angiography (MRA) were also performed (Figure 1). These revealed acute paramedian thalamic ischemic lesions extending to the rostral midbrain (asymmetrically, mainly on the left side). The imaging showed no evidence of cerebral venous occlusion, infiltrative neoplasm, severe infectious and inflammatory lesion, or a large embolus at the basilar tip, with stroke in the posterior circulation. MRA showed patency of the basilar and vertebral arteries, a normal appearance of the left P1 arterial segment and left PCA, and a right-sided full FPCA. Our 1.5-Tesla MRI device failed to visualize the TPAs; the left AOP was just presumed.

He recovered from a coma after 4 days and exhibited a slow, progressive evolution. Initially, he presented with severe alternating (superior) oculomotor hemiplegia (Weber syndrome), with left-sided oculomotor nerve palsy, a drooping eyelid and fixed-width pupil pointed down and out, diplopia, and dysarthria associated with contralateral severe ataxic hemiparesis.

Based on clinical and neuroimaging findings, the positive diagnosis was acute ischemic stroke in the territory of the left AOP. The clinical spectrum of the AOP infarct was outlined in the frame of a “thalamopeduncular" syndrome, associated with the typical symptoms of bilateral paramedian thalamic infarcts (confusion and coma), accompanied by with oculomotor disturbances, contralateral hemiplegia, and cerebellar ataxia.

After the acute episode, he was admitted on the neurorehabilitation department. He clinically manifested a paramedian midbrain syndrome, combining the previously described left-sided oculomotor impairment with moderate right-sided ataxic hemiparesis without hemianesthesia, tremor, dysmetria, dysarthria, and depression. Repeated EKG and blood tests and a transthoracic echocardiogram, respectively, did not reveal pathological aspects.

The diagnosis was established retrospectively, after a delay of 20 hours, too late for thrombolytic management. He initially received anticoagulant therapy (heparin for 3 weeks in the acute stroke department), followed by a novel oral anticoagulant for another 5 weeks during rehabilitation. He was discharged with small doses of aspirin, up to six months, as secondary prophylaxis. Statins were not administered, either in the acute or during the subacute stage.

He had a good evolutive trend and was discharged with a modified Rankin score (mRS) 3. Psychological evaluation emphasized a marked improvement of his masked depression and augmentation of the Mini-Mental State Examination (MMSE) score (from 23 to 29/30).

He completely changed his lifestyle, with abstinence from both tobacco and cannabis, and continued the rehabilitation program as an outpatient. He exhibited favorable outcomes, with no vascular recurrence.

Four months after the acute stroke he achieved a mRS 2 and was slightly disabled and still unable to carry out all previous activities, especially professional ones (driver). Most symptoms abated, except for slight visual blurring, diplopia, and residual left third cranial nerve palsy.

Favorable neurological results were consistent with repeated neuroimaging tests for control. Contrast-enhanced MR angiography remained unchanged. MRI showed no acute recurrences, but only small residual lacunae.

## 3. Discussion

The case reported herein depicts a peculiar association of two vascular variants of the posterior cerebral circulation: a right-sided FPCA and an inferred AOP emerging from the precommunicating segment (P1) of the left PCA, complicated with ischemic stroke, with fluctuations in consciousness [19], and reversible state of coma [20–24], in a complex chronic toxicological context, and a challenging etiopathogenic diagnosis.

Clinical findings and brain imaging have estimated Percheron infarct pattern in 0.1−2.0% first-ever acute cerebral infarctions [22, 25–27] and in 4% to 18% of all thalamic ischemic strokes [21, 27], respectively.

There is no consensus regarding the real prevalence of this uncommon artery. Percheron stated that almost one-third of human brains present this normal variant [7, 8]. Punctilious morphology studies focusing the diversity of the TPAs indicated that AOP frequency across relevant cadaveric studies performed on unselected adult brains was variable (7.0–11.7%, Table 1) [7–17].

Branching patterns of the TPAs emerging from the P1 segment have variable anatomic and radiologic aspects [7–18]. Microvascular investigations classified TPAs into five different types, schematically represented in Table 2.

**Figure 2:**
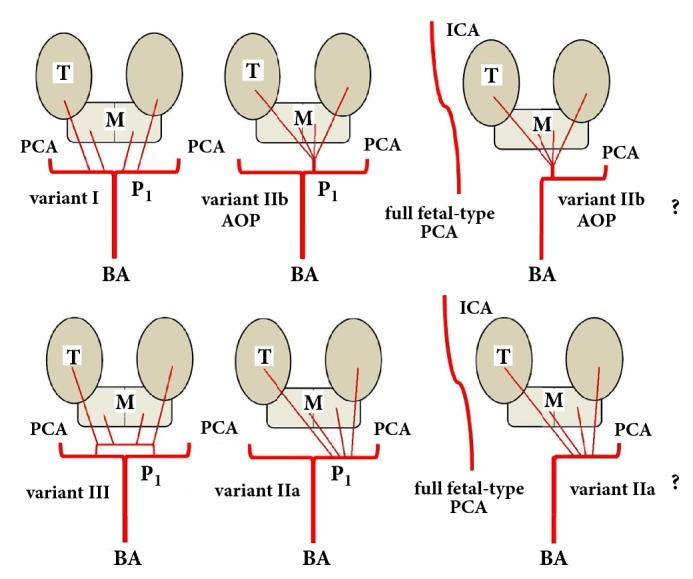
Possible variations involving paramedian perforating thalamic-mesencephalic arterial supply (according to Percheron's description) [7, 8].** I**. The most common: many small perforating arteries arising bilaterally, from P1.** IIb**. The artery of Percheron: a single (asymmetrical) common trunk, arising from one P1 arterial segment.** IIa**. Multiple branches emerging (asymmetrically) from one P1 arterial segment.** III**. An arterial arcade is bridging the P1 segment of both PCAs, and the perforating arteries are arising from this arterial shunt.** The actual case report**: uncommon association of full fetal-type PCA (originating directly from the internal carotid artery) with Percheron's arterial variant. It was not possible to specify the particular anatomical arterial disposal (type IIb or IIa). (T, thalamus; M, mesencephalon; BA, basilar artery; PCA, posterior cerebral artery; P1, first arterial segment of the PCA; AOP, artery of Percheron; ICA, internal carotid artery.)

In case of marked hypoplasia of a single P1 arterial segment or its complete absence (full FPCA), TPAs originate from the contralateral side and cross the midline to supply the medial aspects of both thalami and the rostral midbrain (Table 2 and Figure 2).

Since Percheron's communication in the seventies [7, 8], only isolated case reports and several short-numbered series have focused on this uncommon vascular variant and its pathology. A personal literature review based on a 1973-2018 PubMed search (key items: Percheron, stroke, P1, fetal PCA) found 76 new case reports, added to the 123 analyzed by Zapella in 2014 [24]. Considering the actual report as number two hundred (since Percheron's “description princeps"), one could consider an approximate incidence of 4,44 new cases described annually in literature.

Eleven microvascular anatomical investigations focused on TPAs were realized on unselected cadaveric specimens. Most studies did not encounter morphometric anomalies of P1 (Table 1). Kaya (2010) found different anatomical variants of the posterior circulation in 10.7% specimens, but not the association of AOP with FPCA [14].

It is difficult to visualize and demonstrate these perforating tiny vessels with conventional vascular imaging techniques. Due to their diameters (mean 0.51 mm, min. 0.125, and max. 0.8 mm [7–17]), thalamoperforating branches emerging from P1 might be imperceptible with a 1.5-Tesla MRI scanner, but a 3.0 -Tesla MRA might detect them [28]. Medical imaging progress might be the explanation for the spectacular rise of new reported cases (mentioned above).

Although AOP cannot always be visualized and its presence is only presumed, failure to visualize the vascular anatomy of the perforating arteries does not exclude their presence. In our patient, MRA investigation failed to objectivity demonstrate the posterior TPAs and the ipsilateral and the cross flow to thalamus and midbrain, respectively, because AOP was occluded (or constricted), and the scanner did not exhibit ultra-performance to detect it. It was not possible to establish which of the two variants of TPAs (IIa or IIb) was “the weak link of the chain” in the etiopathogenesis of stroke (Figure 2).

Most significative case series of thalamic strokes were analyzed by Lazzaro (2010) [27], Jiménez Caballero (2010) [26], Song (2011) [29], Arauz (2014) [30], Förster (2014) [31], and Xu (2017) [32]. There were described four clinical and radiological topographic patterns of infarcts: bilateral paramedian thalamic with midbrain involvement (43%), as in the present case report; only bilateral paramedian thalamic without midbrain lesions (38%); bilateral paramedian thalamic associated with involvement of the anterior thalamus and midbrain (14%); and bilateral paramedian thalamic and anterior thalamus infarction, without midbrain involvement (5%) [27].

Matheus and Castillo (2003) [33] have proposed the following paradigm: in case of bilateral thalamic and midbrain infarctions, AOP occlusion should be considered as the main diagnosis.

Förster (2014) [31] studied a database of 600 thalamic infarcts recorded over a decade and selected 48 bilateral thalamic strokes. In 3 subjects (6.3%) he mentioned the association of AOP with FPCA. He pointed out that patients with hypoplastic/absent P1 segments were more likely to have exclusively bilateral paramedian thalamic lesions (P <.001).

To the best of our knowledge, the present case (uncommon vascular association of full FPCA and AOP stroke) might be the fourth one described in the literature.

The morphology and hemodynamic parameters of the perforating arteries in the interpeduncular fossa are strongly influenced by the structure of the circle of Willis [1–6, 34–36].

When an AOP is suspected, consideration should be given to the possibility of other rare anatomical variants of the posterior circulation that may be present [34].

Presence of a fetal-type PCA might also predispose to other strokes because it arises directly from the ipsilateral internal carotid artery and therefore has an established role in extensive cerebral infarction pathogenesis [1, 5, 6, 34–36]. The (full) fetal pattern of PCA hinders the subject`s capacity to build leptomeningeal anastomosis interconnecting the anterior and posterior circulatory systems. By means of arterial spin labeling Barkeij Wolf (2016) [36] quantified cerebral blood flow and demonstrated interhemispheric asymmetry with decreased perfusion in the posterior circulation tributary to the unilateral FPCA.

Etiopathogenesis of this stroke was highly complex and heterogeneous and remained unclear. Extensive, but not exhaustive, investigations have been made to exclude most causes of ischemic stroke in this young adult.

Under advanced and standardized clinical workup in Germany, the detection rate of potential embolic sources was found in 54.2% of bilateral thalamic stroke [31].

The subject abused tobacco smoking, which is known to have a clear, dose-response causal link with stroke [37]. Local thrombosis could not be definitely excluded, because the MR scanner did not exhibit ultra-performance to visualize AOP, and the arteriole was either occluded or constricted.

The absence of atheromatous plaques in the posterior circulation (patency of the vertebral arteries, basilar, and left PCA) and the lack of clotting disorder have argued against an arterial embolization. No obvious clinical source of embolus such as deep venous thrombosis or paroxysmal atrial fibrillation was found.

Cardiogenic embolism could not be eliminated with certainty. In chronic marijuana users smoking can trigger cardiac tachyarrhythmias/paroxysmal atrial fibrillation [38], even with no other identifiable triggers and normal echocardiography. “Embolism not guilty” could not be asserted with certainty because the early hours of the ischemic event have remained an electrophysiopathological “terra incognita”.

Transthoracic echocardiogram was negative for patent foramen ovale related stroke [28, 39, 40]. The etiological study had some weaknesses: bubble contrast echocardiography and contrast transcranial Doppler were not performed, and transesophageal echocardiography (the “golden diagnostic method”) was not available. All were recommended as future investigations and mandatory for an analytical diagnostic procedure.

Knowing the traditional aphorism, “absence of evidence is not evidence of absence", analyzing the “pros and cons" mentioned below, and the fact that embolism has been criticized as the most common AOP occlusion mechanism [19, 27, 28, 38, 41], the reported case might be suspected for embolic stroke of undetermined source [42–44].

AOP infarction reflects well the aphorism: “time is brain". Sometimes, early recognition and optimal time for thrombolytic therapy could be missed. Even more, AOP stroke with delusive normal initial MRI aspects was described in literature [45].

In the diagnostic algorithm the patient underwent a cerebral CT scan (not CT angiography) as first-line imaging procedure. Due to the severity of clinical symptoms MRI was postponed, and not performed at the same time after onset of symptoms; the diagnosis was established too late to receive proper thrombolytic treatment.

A review of the literature focusing on the current therapeutic strategies in emerging AOP occlusion indicated intravenous heparin and thrombolysis with tissue plasminogen activator, as effective first-line treatment options, followed by long-term anticoagulants, whereas in nonemergent cases, without mesencephalic involvement, rehabilitation and continuous monitoring could be the option [46].

Evaluating novel oral anticoagulants versus aspirin, anticoagulants are likely to reduce recurrent brain ischemia more effectively than are antiplatelet drugs, in patients with previous suspected embolic strokes of undetermined source [42–44]. The therapeutic flow in our patient was represented by heparin, followed by a nonvitamin K anticoagulant and then a low dose of aspirin at discharge.

Presence of cognitive-behavioral disorders (memory loss, depression), possible neurological side effects, therapeutic options for polyvitamins and antioxidants (coenzyme Q10, vitamin E), and the absence of an atherosclerotic plaque to be stabilized were the arguments against statins inclusion in the therapeutic arsenal.

Cannabis-related stroke is not a myth, and cannabis consumption should be considered a risk factor for inducing ischemic stroke [40, 47–49]. The case reported a chronic marijuana and tobacco abuse, with a clear time-event related stroke after cannabis smoking (a few hours preceding the cerebral drama). The mechanism by which cannabis may cause cerebral infarction is not completely understood; a drug-induced cerebral angiopathy or a multifocal reversible cerebral vasoconstriction syndrome might be incriminated as etiopathogenetic mechanisms in predisposed chronic abusers [47–49]. In the reported case, the TPAs have not been detected and MRA did not demonstrate evidence of reversible cerebral vasospasm in the posterior circulation, so the “culprit" remained obscured.

Some short-numbered series of AOP infarcts analyzed the correlations between clinical-radiological aspects and outcomes and emphasized that associated midbrain involvement, greater infarct volume, or its hemorrhagic transformation had unfavorable outcomes [29, 30]. Arauz (2014) found that only 25% patients with bilateral paramedian thalamic and mesencephalon extension had good outcomes (mRS score ≤ 2) after a mean follow-up of 55 months [30].

The disciplined reevaluation of his lifestyle and informed decision to stop using tobacco and cannabis led to favorable outcomes. Four months after the acute stroke, the subject showed no dysarthria and motor or coordination deficits and achieved a mRS 2.

A good understanding of the clinical-radiological features of AOP infarction and standardized clinical workup in stroke units that use technology and adequate image performance are essential for early diagnosis and prompt therapeutic intervention.

## Figures and Tables

**Table 1:** Summary of microvascular anatomic investigations of the posterior vascularization and reported incidence of AOP associated or not with other variants in the circle of Willis.

Author, year	Number of brain specimens	Average number of TPAs/brain	Frequency of AOP (%)	P1 segment (hypoplastic or absent)
Grochowski, Maciejewski, 2017 [9]	13	5.8	0%	Not reported
Djulejić, 2015 [10]	12–16	2.2	0%	Not reported
Griessenauer, 2013 [11]	25	Not available	12%	0% (specified)
Kocaeli, 2013 [12]	34	8.5	11,7%	Not reported
Parraga, 2011	35	6	Not reported	Not reported
Park, 2010 [13]	26 (158)	7,2	11,5%	Not reported
Kaya, 2010 [14]	14	6.8	11,7	Not reported
Pai, 2007	25	4	Not reported	Not reported
Uz, 2007 [15]	15	4	7%	Not reported
Cosson, 2003	12	7.5	Not reported	Not reported
Rassi, 1992	30	3.2	Not reported	Not reported
Caruso, 1990	50	8.2	Not reported	Not reported
Marinkovic, 1986 [16]	33	4	Not reported	Not reported
Pedroza, 1986 [17]	28	1-5	10,7%	Not reported
Lang, Bruner, 1978	50	Not available	8%	Not reported
Zeal, Rhoton, 1978	25	5.4	Not reported	Not reported
Saeki, 1977	50	8.2	8%	hypoplastic P1

AOP, artery of Percheron. Data are modified and adapted after Grochowski (2017) [9], Griessenauer (2013) [11], Kocaeli (2013) [12], and Park (2010) [13] and are chronologically arranged.

**Table 2:** Variable branching patterns and origin of the TPAs, emerged unilaterally or bilaterally from the P1 arterial segment.

**Type I** (Park 38.5%)	

TPAs bilaterally, multiple	TPAs bilaterally, multiple

**Type II** (Park 26.9%)	

TPA unilateral, single	TPAs contralateral, multiple

**Type III** (Park 19.2%; *Percheron 40%*)	

TPA single	TPA single

**Type IV **(variant **IIb**,** AOP**) (Park 11.5%; *Percheron 33%*)	**Contralateral P1** segment: present/ absent (full FPCA)** /** hypoplastic

TPA arises unilaterally (from one P1, as a **single** unpaired trunk, AOP)	**−**

**Type V **(variant **IIa**) (Park 3.8%; *Percheron 7%*)	**Contralateral P1** segment: present/ absent (full FPCA)/hypoplastic

TPAs arise unilaterally (from one P1, as **multiple** branches)	**−**

*Percheron's* **variant III (C)**: an ***arterial arcade*** is bridging P1 segments of both PCAs; the perforating branches arise from this ***arterial shunt***.	

TPAs, thalamoperforating arteries. P1, proximal arterial segment of the PCA (from the top of the basilar artery, to the PCoA). **IIa** and **IIb**, arterial variants of vascularization of the thalamus and midbrain described by Percheron. In brackets Park's [13] and *Percheron's *[7, 8] data are mentioned, respectively.

## Data Availability

The clinical data and imagery used to support the findings of this study are included within the article.

## Abbreviations

AOP: Artery of Percheron
CT: Computed tomography
EKG: Electrocardiogram
FLAIR: Fluid-attenuation inversion recovery images
FPCA: Fetal posterior cerebral artery
GCS: Glasgow coma scale
ICA: Internal carotid artery
MMSE: Mini-Mental State Examination
MRA: Magnetic resonance angiography
MRI: Magnetic resonance imaging
mRS: Modified Rankin scale
PCA: Posterior cerebral artery
PCoA: Posterior communicating artery
P1: Proximal arterial segment of the PCA (from the top of the basilar artery to the PCoA)
SCA: Superior cerebellar artery
TPA: Thalamoperforating artery.
